# The Impact of Socioeconomic Status, Surgical Resection and Type of Hospital on Survival in Patients with Pancreatic Cancer. A Population-Based Study in The Netherlands

**DOI:** 10.1371/journal.pone.0166449

**Published:** 2016-11-10

**Authors:** Margijske H. G. van Roest, Maaike A. van der Aa, Lydia G. M. van der Geest, Koert P. de Jong

**Affiliations:** 1 Department of Hepato-Pancreato-Biliary Surgery & Liver Transplantation, University Medical Center Groningen, University of Groningen, Groningen, The Netherlands; 2 Department of Research, Netherlands Comprehensive Cancer Organisation (IKNL), Utrecht, The Netherlands; University of South Alabama Mitchell Cancer Institute, UNITED STATES

## Abstract

The influence of socioeconomic inequalities in pancreatic cancer patients and especially its effect in patients who had a resection is not known. Hospital type in which resection is performed might also influence outcome. Patients diagnosed with pancreatic cancer from 1989 to 2011 (n = 34,757) were selected from the population-based Netherlands Cancer Registry. Postal code was used to determine SES. Multivariable survival analyses using Cox regression were conducted to discriminate independent risk factors for death. Patients living in a high SES neighborhood more often underwent resection and more often were operated in a university hospital. After adjustment for clinicopathological factors, risk of dying was increased independently for patients with intermediate and low SES compared to patients with high SES. After resection, no survival difference was found among patients in the three SES groups. However, survival was better for patients treated in university hospitals compared to patients treated in non-university hospitals. Low SES was an independent risk factor for poor survival in patients with pancreatic cancer. SES was not an adverse risk factor after resection. Resection in non-university hospitals was associated with a worse prognosis.

## Introduction

Despite advances in knowledge concerning risk factor reduction and improvements in early detection and treatment of several cancers, socioeconomic inequalities persist in incidence and survival[1–5]. Low socioeconomic status (SES) has proven to be an important risk factor for developing upper aerodigestive tract cancer[6]. It is also a risk factor for poor survival in patients with cancer of lung[7], stomach[8], and breast[9], as well as hepatocellular carcinoma[10]. Furthermore it is associated with late presentation and recurrence in colorectal cancer[11].

Like in other countries, mortality rates from pancreatic cancer in the Netherlands remain high. The 1-year survival is 18% and only four percent of the patients is alive after five years (period 1989–2010). The incidence of pancreatic carcinoma is slightly increasing in the Netherlands; 8.9/100.000 persons in 1989 and 9.4/100.000 persons in 2015(ESR: European standard Ratio)[12]. Pancreatic resection is the only potentially curative treatment, but only a minority of the patients with pancreatic cancer are eligible for surgery.

Some studies showed that pancreatic cancer patients of low SES neighborhoods were less likely to receive surgical resection[5], chemotherapy[13], or radiotherapy[2], and had significantly higher perioperative and long-term mortality rates than patients with higher SES[2,14]. This may be the result of poorer access to health care for people with low SES, which results in delayed diagnosis and inferior treatment. Although we would not expect this to be the case in the Netherlands, as the obligatory insurance coverage should prevent inequalities in health care access, a higher resection rate was found in a study about stomach cancer in the Netherlands[8]. We therefore aimed to evaluate the patients who underwent pancreatic resection. Since there is a known relationship between hospital volume and mortality after resection of pancreatic cancer[15,16], we investigated whether patients from higher SES groups were more often referred to high-volume university hospitals for treatment. To test these hypotheses we performed a nationwide study in the Netherlands using the population-based database from the Netherlands Cancer Registry.

## Methods

### Cancer registration

All patients diagnosed with pancreatic carcinoma between January 1^st^, 1989 and December 31, 2011 (n = 34,757) were selected from the population-based Netherlands Cancer Registry, which contains data on all patients newly diagnosed with cancer in the Netherlands. The cancer registry obtains notifications from PALGA (Pathologisch Anatomisch Landelijk Geautomatiseerd Archief), the nationwide network and registry of histopathology and cytopathology in the Netherlands. Additional sources are the national registry of hospital discharge, which in general accounts for another eight percent of new cases, and–in a minority of cases- radiotherapy institutions. Information on patient characteristics and tumor characteristics such as subsite (International Classification of Diseases for Oncology (ICD-O-3)[17], histology, stage (Tumor lymph Node Metastasis (TNM) classification[18]), and grade, are obtained routinely from the medical records. In patients who underwent resection (14% of the total study group) stage was based on pathological TNM (I,II,III,IV). In the 86% unresectable patients, the clinical stage was based on clinical TNM or the one-dimensional Extent of Disease was used. These were combined into one variable with 4 categories; 1) ‘local’ = tumor irrespective of size but confined to the pancreas, classification T1 or T2 (TNM 6^th^ and 7^th^ edition); 2) ‘beyond pancreas’ = tumor extension beyond pancreas, classification T2 (according to TNM classification 4^th^ and 5^th^ edition), or T3 or T4, or positive lymph nodes; 3) metastasized disease, M1; and 4) unknown stage. The site of the tumor in the pancreas is reported as head (ICD-O C25.0), not head (ICD-O C25.1–78) and “not otherwise specified/overlapping” (C25.8–9).

The type of hospital in which the pancreatic resection was performed was categorized into: (1) university hospitals (n = 8), (2) non-university teaching hospitals (n = 46) and (3) non-university non-teaching hospitals (n = 46; classification in 2010). A teaching hospital is defined as any hospital which provides medical training to surgical residents to become board-certified surgeons. If the hospital of surgery was not registered, the hospital of diagnosis was assumed to be the hospital of surgery.

The quality of the data in the Netherlands Cancer Registry is high, due to thorough training of the administrators and computerized consistency checks at regional and national levels and completeness is estimated to be at least 95%[19]. Follow-up of vital status of all patients was calculated as the time from the date of diagnosis to the date of death or to January 1^st^, 2013. The information on vital status was initially obtained from municipal registries and from 1995 onwards from the nationwide municipal population registries network.

### Socioeconomic status (SES) scores

Postal code at time of diagnosis was used to determine SES. SES scores are available for each of the 4,002 four-digit postal code neighborhood in the Netherlands. SES scores were calculated in 2006, and these formed the basis for the current analysis. The mean number of inhabitants was 4080 per postal code in 2006. SES scores are provided by the Netherlands Institute for Social Research (Sociaal Cultureel Planbureau) and based on the following items which were collected per six-digit postal code: 1) mean annual income per household, 2) the percentage of households with a low income and 3) the percentage of households with a low education[20].

SES was divided into three groups based on the delivered rank numbers: low (1^st^-3^rd^ deciles, n = 10,294), intermediate (4^th^-7^th^, n = 13,775) and high (8^th^-10^th^, n = 10,274) SES.

### Statistical analyses

Associations between SES of neighborhood, stage of disease, localization of the tumor, histological grade of the tumor and treatment were analyzed by Chi-square analysis and calculation of 95% confidence intervals (95%CI). Association between SES and age at diagnosis were analyzed by one-way Anova analysis.

Overall 5-year survival rates were calculated. Cox’ regression models were used to compute multivariable rates (Hazard Ratio = HR, forward procedure) and 95% confidence intervals (95%CI). Statistical analysis was performed with Stata version 12.

## Results

In the period 1989–2011 34,757 patients were diagnosed with pancreatic cancer in the Netherlands. The mean age at diagnosis was 70 years (range 15 to 101 years). Most patients were diagnosed with metastatic disease (45%).

Table 1 shows differences in patient and tumor characteristics between different SES groups. The low SES group is characterised by more females, slightly older age, and less frequent resection of the tumor. Of note, 10% of the patients living in high SES neighborhoods underwent resection, whereas in the low SES neighborhoods this was 9% (p = 0.006, Table 1). Patients with higher SES were treated with adjuvant chemotherapy more often than patients with lower SES (22% versus 18%, p = 0.02).

**Table 1:** Distribution of individual characteristics of patients with pancreatic cancer in the Netherlands across different socioeconomic groups. (n = 34,757).

		Low SES		Intermediate SES		High SES		
		%	95% CI	%	95% CI	%	95% CI	p-value
**Gender**	Male	48	47–49	50	49–51	50	49–51	0.02
	Female	52	51–53	50	49–51	50	49–51	
**Age**	<30	0.1	0.0–0.1	0.1	0.0–0.1	0.1	0.0–0.1	0.006
	30–44	1.9	1.6–2.2	2.0	1.7–2.2	2.2	2.0–2.5	
	45–59	16	15–17	17	16–17	18	–17-18	
	60–74	43	42–44	44	43–45	44	43–45	
	75+	39	38–40	37	36–38	37	36–38	
**Tumorlocalization**	Head	68	67–69	69	68–69	68	67–68	0.009
	Non-head	18	18–19	18	17–19	20	19–21	
	Overlapping lesion/nos	13	13–14	14	13–14	13	12–13	
**Differentiation**	Well	3.06	2.7–3.4	3.2	2.9–3.5	2.9	2.5–3.2	0.015
	Moderate	10	10–11	11	10–11	11	11–12	
	Poor	10	10–11	11	11–12	11	28–30	
	Undifferentiated	0.7	0.60.9	0.7	0.5–0.8	0.7	0.5–0.8	
	Unknown	76	75–77	74	74–75	74	73–75	
**Stage**	local	14	14–15	14	13–14	13	12–13	0.02
	Beyond pancreas	28	27–29	28	28–29	29	28–30	
	metastatic	45	44–46	45	44–46	45	44–46	
	Unknown	13	12–13	13	12–13	14	13–15	
**Resection**	No	91	90–10	90	90–91	90	89–90	0.006
	Yes	9	9–10	10	9–10	10	10–11	

The highest volume of operated patients was observed in university hospitals; eight hospitals performed 1,198 resections (= 35% (34–37% 95%CI)), as compared to 1,350 resections (= 40% (38–41% 95%CI)) in 46 non-university teaching hospitals and 833 resections (= 25% (23–26% 95% CI)) in 41 non-university non-teaching hospitals.

Table 2 summarizes the prognostic factors in all patients presenting with pancreatic cancer. Favorable prognostic factors in multivariable analysis were a medium and high SES and resectable tumors, while adverse prognostic factors were older age, non-head tumor localization, tumor differentiation other than well differentiated, and TNM stage I.

**Table 2:** Univariable and multivariable analysis of variables in relation to Hazard Ratio (HR) of pancreatic cancer patients in the Netherlands, period 1989–2011 (n = 34,757).

Variables			Univariable		Multivariable	
		%	HR	95% CI	HR	95% CI
**SES**	Low	30	1	Reference	1	Reference
	Medium	40	0.96	094–0.99	0.96	0.94–0.99
	High	30	0.92	0.90–0.95	0.93	0.90–0.95
**Age**		70(median)	1.0	1.0–1.0	1.02	1.01–1.02
**Gender**	Men	49	1	Reference	1	Reference
	Women	51	0.99	0.98–1.02	0.96	0.94–0.98
**Localization**	Head	68	1	Reference	1	Reference
	Non-head	19	1.3	1.2–1.3	1.1	1.07–1.13
	NOS/Overlapping lesion	13	1.5	1.4–1.5	1.20	1.16–1.24
**Differentiation**	Well	3	1	Reference	1	Reference
	Moderate	11	1.1	1.1–1.2	1.	1.13–1.30
	Poor	11	1.7	1.6–1.8	1.5	1.43–1.64
	Undifferentiated	1	2.9	2.5–3.4	1.95	1.69–2.25
	Unknown	75	1.9	1.8–2.0	1.21	1.43–1.29
**Stage**	local	14	1	Reference	1	Reference
	Beyond pancreas	28	1.0	0.9–1.0	1203	1.15–1.24
	metastatic	45	2.4	2.3–2.5	2.29	2.21–2.37
	unknown	5	1.5	1.5–1.6	1.31	1.26–1.37
**Resection**	No	90	1	Reference	1	Reference
	Yes	10	0.3	0.3–0.3	0.43	0.41–0.45
**Adjuvant chemotherapy**	No	80	1	Reference	Not included in multivariable analysis	
	Yes	20	0.3	0.3–0.3		

CI: Confidence Interval.

Table 3 shows the prognostic factors in 3,381 patients who underwent a resection. On multivariable analysis adverse prognostic factors were: operation in other than university hospitals and poorly differentiated tumors. Patients with TNM stage I disease had a more favorable prognosis compared to patients with other stages of disease. Of note, whereas SES was a risk factor in the whole group of pancreatic cancer patients, this was not the case anymore in the group of patients who underwent a resection (univariable analysis SES Low (reference) HR: 1; SES Medium HR1.04 (95% CI 0.9–1.1; SES High HR 0.98 (95% CI 0.89–1.07).

**Table 3:** Univariable and multivariable analysis of variables in relation to Hazard Ratio (HR) of pancreatic cancer patients who underwent resection in the Netherlands, period 2005–2011. (n = 3,381).

			Univariable		Multivariable	
		% of patients	HR	95% CI	HR	95% CI
**Type of hospital**	University	35	1	Reference	1	Reference
	Non-university, teaching	40	1.2	1.1–1.3	1.2	1.13–1.33
	Non-university, non teaching	25	1.3	1.-149	1.5	1.33–1.61
**Age**		65(median)	1.01	1.0–1.02	1.01	1.00–1.02
**Gender**	Men	54	1	Reference	1	Reference
	Women	46	0.95	0.9–1.0	1.00	0.93–1.08
**Differentiation**	Well	11	1	Reference	1	Reference
	Moderate	44	1.3	1.2–1.5	1.3	1.12–1.44
	Poor	29	1.8	1.6–2.0	1.7	1.5–2.0
	Undifferentiated	0.3	1.3	0.6–2.6	0.9	0.5–1.9
	Unknown	17	1.0	0.9–1.2	1.1	0.96–1.27
**Stage (TNM)**	I	26	1	Reference	1	Reference
	II	44	1.5	1.9–2.8	1.5	1.35–1.63
	III	22	1.9	1.9–3.3	1.8	1.66–2.05
	IV	5	2.5	2.1–5.0	2.5	2.14–2.99
	Unknown	2	1.0	0.8–1.3	1.1	0.86–1.42
**Adjuvant chemotherapy**	No	80	1	Reference	1	Reference
	Yes	20	0.7	0.6–0.8	0.7	0.64–0.78

## Discussion

The main findings of this nationwide study in patients with pancreatic cancer are that low SES is an independent risk factor for poor survival, whereas in pancreatic cancer patients who underwent resection, SES is not a risk factor. These results confirm findings in other studies in which pancreatic cancer patients with lower SES tend to have worse survival and are less likely to receive adequate treatment (Table 4). In the two series reporting on patients who underwent resection the results are contradictory: the Lim paper [21] (n = 396 patients) describes lower survival in the low SES group, whereas in the Kuhn paper [22] (n = 117 patients) no effect was found.

**Table 4:** Overview of studies describing pancreatic cancer survival in relationship with a low socioeconomic status.

Author	Year of public-cation	Number of patients	Tumor	% resectionadeno-carcinoma	Influence of low SES				
					Survival (MV)	Surgery	Chemo-therapy	Radio-Therapy	Other
Blot[23]	1978	United States survey	PaC	-	None	-	-	-	Higher pancreatic mortality in urban residents
Janes[24]	1996	16,942	PaC	8.8	-	↓	↓	↓	More resections and lower postoperative mortality if treated in high volume center or in teaching hospital
Krzyzanowska[25]	2003	1,696	LA-PaC	-	-	-	↓	↓	More cancer directed therapy if treated in teaching hospital
Lim[21]	2003	396	PaC	Only patients who underwent resection	↓	↓	↓	↓	Better survival if surgery in teaching hospital
Van Oost[26]	2006	1,130	PaC	11	-	-	-	-	Low SES: less referred to university hospital
Cress[14]	2006	10,612	PaC	15.8	↓(UV)	↓	-	-	
Zell[27]	2007	24,735	70.1% PaC	11.8	↓ (UV)	↓	↓	↓	
Le[28]	2008	15,296	66.6% PaC 0.3% IPMN	12	None*	↓	↓	↓	
Kuhn[22]	2009	117	PaC	Only patients who underwent resection	None	-	-	-	
Cheung[2]	2010	16,104	PaC	18.8†	↓	↓	↓	↓	Low SES: younger at diagnosis
Seyedin[29]	2012	5,908	PaC	No data	-	↓	-	-	
Cheung[30]	2013	58,747	PaC	16.2	-	↓	-	-	
Bernards[13]	2014	1,494	PaC**	-	-	-	↓	-	
Enewold[31]	2015	977	PaC	22.1	None	None	-	-	Low SES: more frequently associated with no treatment
Wolfson[32]	2015	2,317	PaC	-	-	-	-	-	Low SES: less patients treated in NCICCC facility∫
Markossian[33]	2015	245	PaC	29	↓	-	-	-	
Shapiro[5]	2015	17,530	PaC	45.4	-	↓	-	-	Lower SES: worse stage at presentation
Present Series		34,757	PaC	14.9	↓	↓	↓	-	

Abbreviations: PaC: pancreatic adenocarcinoma; LA-PaC: locally advanced pancreatic cancer; IPMN: intraductal papillary mucinous neoplasms;

^-^: no data;

^↓^: decreased;

MV: multivariable analysis;

UV: univariable analysis;

*: only studied for IPMN tumors;

^†^: patients treated surgically (not only resection);

NCICCC: National Cancer Institute Comprehensive Cancer Center;

^∫^: patients treated in a NCICCC had better survival and were presented with lower stage of disease;

**: only patients with metastatic disease.

Among the variables which are significantly different in the three SES groups–gender, age, tumor localization, differentiation, stage and resection—we consider only resection a clinical relevant variable, because this has a major impact on outcome. In this study, patients with low SES less often underwent resection and had therefore more often an unknown stage than patients with high SES. After adjustment for confounding factors, SES remained an independent prognostic variable. However, in patients with resectable tumors, SES was no longer a relevant variable for survival. This was also found in a case series of 117 patients in Germany in which the SES was obtained from individual patients[22].

We found that patients with higher SES were more often operated in a university hospital. Furthermore, resection for pancreatic cancer in a non-university (teaching or non-teaching) hospital was associated with an increased risk of dying as compared to resection in a university hospital. University hospitals have higher volumes of pancreatic resections, because of referral patterns for this type of surgery. The lower volumes in most of the non-university hospitals might be a part of the explanation for the worse outcome, because low volume is a well-known risk factor for poor outcome in various surgical procedures including pancreatic resections [15,16,24]. For example, a study in a population of patients with periampullary cancer in the Southern part of the Netherlands also revealed that patients who underwent resection in a university hospital had a better three-month survival as compared to patients treated in a low volume hospital (performing less than five resections a year) in the region[26]. However, apart from differences in patient selection of patient referral, other explanations might be differences in staff-to-patient ratios, financial resources, more easy access to specialized diagnostic and treatment possibilities, and higher qualified intensive care units[34].

Health disparities originate in the complex interplay of patient, health-care provider and institutional factors. Major determinants of survival are tumor characteristics[35], and the presence of disseminated disease[36]. Other factors like psycho-social and bio-behavioral influences[37], environmental influences, and income-related lifestyle factors may play a role in cancer pathogenesis[5,38,39]. Patients of lower SES probably have more associated comorbidity[40] and additionally may lack the knowledge to comprehend the complex details of their diagnosis, may not be well informed about the possible improved outcome at university hospitals and may lack social support and structure[2]. Data from a study in the United States showed that patients with pancreatic carcinoma with lower SES were treated more frequently in low volume hospitals, compared with patients with higher SES. However, when patients from lower SES were treated in a high volume and or teaching hospital, they still had increased perioperative mortality and shorter median survival[2]. In a recently published paper on patients after pancreaticoduodenectomy, we could demonstrate that in the Netherlands 30-day mortality was 4.6% and 90-day mortality 7.8%. In that paper, describing only patients who underwent resection, SES was not predicting peri-operative death, neither 30-day mortality nor 90-day mortality. Thus, we extrapolate that postoperative mortality and SES are not predictors of cancer survival.[41]

In our study, patients from higher SES neighborhoods were more likely to receive adjuvant chemotherapy. We cannot exclude the likelihood that patients who received adjuvant therapy were less ill and thus judged to be better candidates for such treatment. Treatment with adjuvant chemotherapy has been introduced in the Netherlands in 2007 after publication of the CONKO-001 study[42]. Therefore only limited data are available precluding a multivariable analysis with adjuvant chemotherapy. Possibly this chemotherapy contributes to better survival in the latest period.

In 40% of patients the presumed diagnosis of pancreatic cancer could not be proven by histology or cytology, because most of the patients with pancreatic cancer are unresectable, which also explains that around 75% of the patients in each SES group has an unknown histological grade.

Limitations of our study are the way of measuring SES and the lack of information on specific referral patterns in relation to SES. We used an ecological measure of SES, which was assigned to each individual patient according to the postal code of residence at the time of diagnosis. Theoretically it is possible that individual people are misclassified and that inferences at the area level do not directly transfer to individuals. In practice, validation studies, however have found that an area-based measure of SES is a good indicator of SES for individuals. Another limitation is the absence of information on comorbidity. Comorbidity may be a factor underlying lower survival in cancer patients with low SES[40].

In conclusion, this study demonstrated that SES was an independent prognostic factor in a nationwide-study of a large cohort of pancreatic cancer patients in the Netherlands. Performing a pancreatic resection is the most important prognostic factor and is more frequently performed in patients with high SES versus patients with low SES. Remarkably, in patients who underwent resection, SES is no longer a risk factor. Resection in university hospitals is associated with the highest chance of survival. With the knowledge of the differences in treatment between patients with different SES, specialists treating patients with pancreatic cancer should make effort to explain the different treatment options to all patients groups clearly and afford referral to a center for pancreatic surgery.
